# Effects of Workplace-Related Factors on the Prevalence of Fibromyalgia among Israeli Kindergarten Teachers

**DOI:** 10.1155/2020/3864571

**Published:** 2020-10-23

**Authors:** Yafa Buskila, Tamar Chen-Levi, Dan Buskila, Giris Jacob, Jacob J. Ablin

**Affiliations:** ^1^Orot Israel College of Education, Rehovot, Israel; ^2^Ben Gurion University of the Negev, Beersheeba, Israel; ^3^Internal Medicine F, Sourasky Medical Center, Tel Aviv & Sackler School of Medicine, Tel Aviv University, Tel Aviv, Israel; ^4^Internal Medicine H, Sourasky Medical Center, Tel Aviv & Sackler School of Medicine, Tel Aviv University, Tel Aviv, Israel

## Abstract

**Background:**

Fibromyalgia syndrome (FMS), a chronic widespread pain disorder, has been associated with various models of stress, including those that are workplace-related. In a previous study, we have documented the significantly increased prevalence of FMS among schoolteachers, as well as correlating symptoms with stressful workplace-related factors. In the current study, we have focused on the specific population of kindergarten teachers and attempted to document both the prevalence of FMS symptoms among this group and the association with stress and symptoms of posttrauma.

**Methods:**

All participants in the study were working as kindergarten teachers in Israel at the time of the study. Participants responded to a questionnaire documenting FMS symptom, which included the widespread pain index (WPI) and symptom severity scale (SSS), which together constitute the suggested American College of Rheumatology (ACR) FMS diagnostic criteria. Additional items on the questionnaire documented work motivation and performance, the occurrence of workplace-related stressful events, and the presence of posttraumatic symptoms.

**Results:**

242 participants were recruited to the current study, including 239 (98.8%) females and 3 (1.2%) males. 62 individuals (25.6%) were found to fulfill ACR FMS criteria. Significant differences in work performance were found between teachers fulfilling FMS criteria compared with those not fulfilling criteria. Thus, FMS-positive teachers reported significantly higher rates of missing workdays, leaving work early, and a lower quality of interaction with children in the kindergarten and with peers and supervisors. Motivation to work was also significantly lower among these individuals. The widespread pain index (WPI) and symptom severity scale (SSS), which together constitute the components of the FMS diagnostic criteria, were positively correlated with both stress and posttraumatic symptoms. In addition, widespread pain, disordered sleep, difficulty with concentration, and other FMS symptoms were strongly correlated with many specific stressful factors at the workplace, including the number of children in the kindergarten, interaction with parents, lack of optimal physical conditions in the classrooms, and various demands on behalf of the educational system.

**Conclusion:**

FMS symptoms were found to be highly prevalent among Israeli kindergarten teachers, at a rate that greatly exceeds the prevalence in the general Israeli population. Stressful work-related events appear to be positively associated with the occurrence of FMS symptoms and may serve as triggers for their development. Healthcare professionals treating individuals engaged in this occupation should be vigilant for the occurrence of symptoms that are clinically associated with FMS and overlapping functional disorders.

## 1. Introduction

Fibromyalgia (FMS), a syndrome clinically characterized by chronic widespread pain and fatigue, is currently considered a prototype of a nociplastic pain [1], i.e., a condition in which chronic pain originates within the central nervous system, rather than being attributable to any peripheral pathology [2, 3]. As such, FMS has attracted a great deal of interest focusing on the mechanisms through which pain processing may be altered from the normal into the pathological and into the possible triggers and predispositions underlying this transformation. In this context, a considerable amount of research has been directed at the association between stress and pain centralization, with many different models of stress being utilized in order to understands this association. Thus, childhood trauma and abuse [4, 5], stress related to war, and natural disasters [6–8] are among such models. Workplace-related stress is a unique model in which stress is ongoing rather than acute and in which a broad variety of measurable factors related to the specific conditions within the workplace may be separately analyzed, in order to evaluate their contribution to work-related stress. While workplace-related bullying has specifically been associated in the past with an increased rate of the development of FMS symptoms [9], work can be highly stressful even without intentional (or indeed unintentional) bullying and abuse. Thus, in a related previous study, we have demonstrated the high prevalence of FMS symptoms among school teachers, as well as identifying specific workplace-related stressors acting as possible triggers in this milieu [10]. In the current study, we have focused on a sample of kindergarten teachers, a group with some rather unique characteristics, and attempted to analyze both the rate of FMS-related symptoms as well as the correlation between specific workplace-related stress and the expression of such symptoms. These findings may be of clinical, theoretical, and occupational significance.

## 2. Methods

The study was conducted as an online survey, distributed to a sample of Israeli kindergarten teachers reached out to by the authors. The study questionnaire, which was formatted as a Google-docs file, documented demographic data, including age, marital status, education, medical history (comorbidities), religion, and years on the job. Subsequent questions addressed workplace-related factors which were considered stressful by the respondents. These included factors such as interaction with supervision officials, interaction with parents, number of children in the kindergarten, composition of children characteristics (special need children), and physical conditions at the workplace (e.g., crowding and lack of air conditioning).

### 2.1. Assessment of Pain and Other FMS-Related Symptoms

The study questionnaire included specific items regarding the presence of a broad spectrum of pain-related symptoms, including headache, back pain, and widespread pain. Additional questions focused on sleep disturbances, fatigue, joint stiffness, paresthesia, swelling, irritable bowel symptoms, anxiety, depression, and difficulty with memory and concentration. These questions were answered on a scale of 1 to 10, with 10 signifying the maximal severity of each symptom. Patients answering in the affirmative regarding the presence of pain were further asked about the time course and chronicity of pain, body distribution of pain, medical investigations and treatments received for pain, and loss of workdays as a result of pain. Participants were also asked to fill out the widespread pain index (WPI) and symptom severity scale (SSS), which together provide the basis for a diagnosis of FMS according to the 2011ACR survey diagnostic criteria [11]. These tools were subsequently used in order to calculate the prevalence of FMS in the study population.

### 2.2. Assessment of Trauma and Posttraumatic Symptoms

In order to assess the possible association of workplace-related stressful events with posttraumatic symptoms in the study population, the questionnaire included the posttraumatic diagnostic scale [12], which we have previously used for the assessment of posttraumatic symptoms in the context of screening for chronic pain and FMS [7]. We subsequently analyzed the correlation between FMS-related symptoms and PTSD symptoms.

### 2.3. Assessment of the Influence of Symptoms on Work Performance and Motivation

In order to evaluate the association between FMS-related symptoms and work performance, participants were questioned regarding their perception of the relationship between their health and their work motivation, work absence, quality of work, relationship with peer workers, and relationship with parents and children.

Participants were asked about a range of work performance related parameters. This included questions about motivation to reach work, attendance, efforts invested in creating student interest, punctuality of arrival, caring for children, self-assessed quality of care, and relations with parents. The results of these items were correlated with FMS symptoms in the whole study group and a dichotomous comparison was subsequently performed between participants fulfilling and not fulfilling FMS criteria.

### 2.4. Assessing the Interaction between Workplace-Induced Stress and FMS Symptoms

In order to evaluate the association between workplace-induced stress conditions and the occurrence of FMS symptoms, participating kindergarten teachers were requested to answer a series of questions, regarding which of the following factors they considered a source of workplace-related stress, on a scale of 1–10: kindergarten supervisor, kindergarten assistants, student parents, size (number of pupils) of classroom, composition of student population (e.g., students with ADHD/ADD and students requiring special education), lack of adequate physical conditions (e.g., lack of air-conditioning), intensiveness of work, multiple demands related to reporting and filling-out forms, multiple educational programs introduced into the kindergarten, demands regarding initiating new programs, home responsibilities conflicting with work-responsibilities, continuing education programs (including after-work-hours programs), competitive feelings regarding meeting educational requirements, preparing and managing kindergarten parties and special occasions and the system's support for kindergarten teachers during times of crisis. The abovementioned stress factors were correlated with FMS-related symptoms including widespread pain, headache, backache, difficulty with memory and concentration, depression, anxiety, and irritable bowel syndrome (IBS) symptoms.

## 3. Statistics

SPSS 21 software was used in all statistical calculations. Using the results of the WPI and SSS, it was possible to determine the proportion of responders who met ACR criteria for a diagnosis of FMS and subsequently to compare FMS-positive and FMS-negative responders regarding the various items covered by the study questionnaire, including work-related items and trauma-related items. Pearson correlation calculations were implemented in order to evaluate correlation between FMS-related symptoms and work-related stress factors, as well as correlations between posttraumatic symptoms and FMS symptoms. Work performance of FMS-positive and FMS-negative responders was compared using a *T*-test calculation.

## 4. Results

Two hundred forty-two participants were recruited to the current study, including 239 (98.8%) females and 3 (1.2%) males. 62 participants met ACR diagnostic criteria for FMS, giving a prevalence of 25.6% for the entire study population. As 99.6% of the study population were Jewish, participants were further categorized according to their self-reported level of religiosity, stratified as “secular” (26.9%), “conservative” (18.2%), “religious” (31.4%), and “ultraorthodox” (Haredi) (20.2%). 66.%% of participants defined themselves as Sephardi Jews, while 30.6% were Ashkenazi Jews. No significant differences were found regarding FMS prevalence among these categories.

In terms of education, out of 242 participants, 163 (67.4%) reported holding a university first degree, while 65 (26.9%) held a second degree. 10 (4.1%) reported having a high school education. No significant differences were found regarding FMS prevalence among these categories.

In terms of the age of participants, out of 242 participants, 34 (14.0%) were aged 20–29, 74 (30.6%) were aged 30–39, 73 (30.2%) were aged 40–49, 53 (21.9%) aged 50–59, and 6 (2.5%) were aged above 60. No significant differences were found regarding FMS prevalence among these categories.

### 4.1. Assessing the Interaction between Workplace-Induced Stress and FMS Symptoms

Table 1 presents correlations between symptoms and stressful factors in the kindergarten teacher population. As can be seen in the table, highly significant correlations were observed between signature FMS symptoms and between many of the work-related stress factors, e.g., between widespread pain and factors such as class-room physical conditions and class composition on the one hand and with factors such as the introduction of novel educational programs and the requirement to initiate such programs on the other hand. Sleep disturbances were also significantly correlated with many of the stressful factors, as shown in the table.

### 4.2. Relationship between FMS Symptoms and Traumatic Events

Table 2 presents the results of this analysis. As shown in the table, strong correlations were found between central FMS features, including the WPI and SSS, and between classical PTSD-related symptoms such as hypervigilance, restlessness, and avoidance. These correlations demonstrate a close association between FMS symptoms and PTSD-related symptoms in the population studied, although causality could not be determined.

### 4.3. Assessing Effects of FMS-Related Symptoms on Work Performance

The results demonstrated significant negative correlations between motivation to come to work with the FS (fibromyalgia score, a composite of WPI and SSS) as well as with symptoms of sleep disturbances, concentration difficulties, IBS symptoms, widespread pain, anxiety and depression. Notably, self-reported evaluation of the connection with kindergarten children, was not significantly correlated with the WPI, SSS or FS.

Table 3 presents the results of this correlation. As shown in the table, teachers who met FMS criteria reported significantly lower level of work motivation, were significantly more likely to leave work early or be absent from work, were significantly less likely to conduct the class room to their own satisfaction, and reported significantly lower levels of caring for their pupils.

### 4.4. Assessing Posttraumatic Symptoms among Teachers with and without FMS

Since FMS may be induced by stress and by traumatic triggers, we attempted to evaluate the difference between teachers who met FMS criteria, compared with those who did not, regarding symptoms of posttrauma.  Table 4 presents the results of a *T*-test comparison between these subgroups. As shown in the table, teachers who met FMS criteria had significantly higher scores on many scales of posttrauma including hypervigilance, involuntary sad thoughts, and reexperiencing traumatic events.

## 5. Discussion

In the current study, we have demonstrated a high prevalence of FMS symptoms among Israeli kindergarten teachers, to scale of 10 times the rate reported in the general Israeli population (which is around 2.5% [13]). Notably, however, the population studied in the current research was almost exclusively female, and thus a more appropriate comparison would be to the prevalence of FMS among Israeli females, which was estimated to be between 5.5 and 7%. Nonetheless, the prevalence reached in the current study is remarkable. In a previous recent study, we have used similar methodology in order to estimate the prevalence of FMS among Israeli teachers occupied in grade schools and high schools [10]. 11.4% of female teachers were found to meet FMS criteria in that study, a finding which in itself was also remarkable. Thus, it is truly challenging to attempt to explain the findings of the current study. In this context, it is worthwhile to return to the basic hypothesis we built upon in designing this study; namely, that work-related stress may play a major role in the etiology of FMS symptoms among kindergarten teachers, and that events experienced on a day-to-day basis throughout their work routine may have a decisive and highly deleterious effect. This hypothesis is obviously supported by results of our previously mentioned research among teachers, but is further bolstered by a multitude of studies which have attempted to tie between FMS and different models of stress, ranging from early childhood trauma and abuse [4, 14], through war and other forms of manmade calamity [7, 15] and indeed culminating in workplace-related stress, such as workplace bullying [9]. Thus, while it remains currently unfeasible to pinpoint the exact contribution of stress to the etiology of FMS, or to precisely delineate the interaction between stress and other etiological factor (most notably, genetic predisposition and epigenetics [16]), most authorities would concur in stating that stress plays a role. What is less obvious, however, is the nature of resilience factors which may make some individuals, and indeed some populations, much more vulnerable than others. Resilience is a complex construct, increasingly recognized as playing an important role in chronic illness in general and particularly in FMS [17]. While resilience has been given different definitions over history and in varying scientific disciplines, internal personal characteristics such as balance, perseverance, self-reliance, and, possibly most importantly, attribution of meaning and purpose to life are among the most important attributes of this concept [18, 19]. Resilience is associated with important neurophysiological aspects, such as the capacity to perform inhibition of the amygdala by the median prefrontal cortex (mPFC), which can protect against the development of PTSD and chronic pain [20, 21]. How might this relate to the population of kindergarten teachers? When addressing the results obtained in the current study, it appears obvious that significant correlations are to be found between symptoms which we recognize as part of the FMS spectrum on the one hand (e.g., widespread pain and sleep disorders) and symptoms specifically related to PTSD (e.g., hypervigilance) on the other hand. These symptoms were also found to be significantly more common among kindergarten teachers who met FMS criteria. Thus, a high level of vulnerability to the deleterious effects of workplace-related stressful events must exist in this population, rendering them susceptible to both FMS and PTSD as a result of events within the kindergarten which would not generally be recognized as traumatic to such an extent. It is tempting to speculate whether such vulnerability may be the counterpart of low levels of resilience and what might cause such an extreme association. While we have not specifically looked at resilience in the current study, it must be pointed out that kindergarten teachers do not generally enjoy a very high level of social recognition or of monetary reward. Teacher stress, a term originally coined by Kyriacou [22], addresses various aspects of the teacher's work-related experience and has subsequently become a focus for extensive research in the fields of education and occupational medicine, drawing attention to the importance of teachers burnout versus resilience [23, 24]. Many particular aspects of work have been identified as contributing to teacher stress. In some cases legislation and politically motivated reforms have cast teachers into a turmoil of rapidly changing demands with the need for rapid acquisition of novel capabilities such as computing, budgeting, and risk management, some of which are far beyond the more traditional role of a teacher [25]. Such changes may cause detrimental lack of security among teachers [26]. Teacher stress may be exacerbated by a multitude of tasks the teacher is called upon to perform [27], through required work beyond work hours [28], lack of discipline among overcrowded pupils [29], and coping with the challenges raised by parents [30]. The deleterious health effects of work-related stress among teachers have frequently been documented in the past [30]. Ill-defined symptoms, which were historically lumped under the headline of “psychoneurotic,” have been described among teachers [31], as well as frank psychiatric symptoms such as anxiety, depression, and suicidal ideation. Not surprisingly, such problems have a significant negative occupational price, leading to low work satisfaction, low attendance, and decreased productivity [32]. Tsai et al., looking specifically at a population of female kindergarten teachers in Hong Kong, found that time management and work-related stressors were the more common sources of stress, while fatigue and emotionally related symptoms were the most common manifestations of stress in this population [33].

Thus, multiple factors, some of which are determined inadvertently by society or by the consequences of resource allocation and some are merely resulting from the rapidly changing requirements of the 21^st^ century kindergarten, may be putting teachers in this setting under extreme work-related stress, without providing the recognition and meaning necessary in order to promote resilience. We need to know much more regarding the mechanisms leading from workplace stressors towards chronic pain and posttrauma. Until then, it is important to recognize the clinical association between these factors, when dealing with individuals engaged in this occupation. It is also important for educational leaders and policy makers to recognize the price that may be paid for excessive workload, insufficient physical resources, and inconsistent work demands.

## Figures and Tables

**Table 1:** Correlation between work-related stress and FMS-related symptoms.

	Kindergarten management	Kindergarten assistants	Parents	Class load	Class composition	Physical conditions	Work intensiveness	Reporting and filling forms	Introduction of educational programs	Required to initiate programs	Work-home conflicts	Continued education	Competition	Crisis support
Sleep disorders	0.255^*∗∗*^	0.176^*∗∗*^	0.284^*∗∗*^	0.217^*∗∗*^	0.257^*∗∗*^	0.245^*∗∗*^	0.311^*∗∗*^	0.237^*∗∗*^	0.245^*∗∗*^	0.239^*∗∗*^	0.270^*∗∗*^	00.157^∗^	0.203^*∗∗*^	0.183^*∗∗*^
Fatigue	0.314^*∗∗*^	0.224^*∗∗*^	0.355^*∗∗*^	0.348^*∗∗*^	0.421^*∗∗*^	0.288^*∗∗*^	0.437^*∗∗*^	0.447^*∗∗*^	0.401^*∗∗*^	0.435^*∗∗*^	0.559^*∗∗*^	0.343^*∗∗*^	0.280^*∗∗*^	0.160^∗^
Joint stiffness	0.189^*∗∗*^	0.121	0.073	0.029	0.122	0.101	0.231^*∗∗*^	0.175^*∗∗*^	0.207^*∗∗*^	0.217^*∗∗*^	0.204^*∗∗*^	0.095	0.200^*∗∗*^	0.184^*∗∗*^
Paresthesia	0.126	0.169^*∗∗*^	0.087	0.064	0.099	0.119	0.195^*∗∗*^	0.103	0.169^*∗∗*^	0.126	0.140^*∗*^	0.145^*∗*^	0.120	0.219^*∗∗*^
Feeling of swelling	0.189^*∗∗*^	0.066	0.160^∗^	0.120	0.176^*∗∗*^	0.187^*∗∗*^	0.238^*∗∗*^	0.174^*∗∗*^	0.174^*∗∗*^	0.208^*∗∗*^	0.167^*∗∗*^	0.120	0.161^*∗*^	0.140^*∗*^
IBS	0.192^*∗∗*^	0.076	0.268^*∗∗*^	0.223^*∗∗*^	0.243^*∗∗*^	0.266^*∗∗*^	0.345^*∗∗*^	0.264^*∗∗*^	0.267^*∗∗*^	0.300^*∗∗*^	0.329^*∗∗*^	0.199^*∗∗*^	0.213^*∗∗*^	0.153^∗^
Anxiety	0.395^*∗∗*^	0.269^*∗∗*^	0.419^*∗∗*^	0.272^*∗∗*^	0.321^*∗∗*^	0.272^*∗∗*^	0.328^*∗∗*^	0.329^*∗∗*^	0.307^*∗∗*^	0.375^*∗∗*^	0.392^*∗∗*^	0.231^*∗∗*^	0.246^*∗∗*^	0.231^*∗∗*^
Depression	0.229^*∗∗*^	0.297^*∗∗*^	0.267^*∗∗*^	0.213^*∗∗*^	0.248^*∗∗*^	0.150^∗^	0.332^*∗∗*^	0.265^*∗∗*^	0.232^*∗∗*^	0.204^*∗∗*^	0.243^*∗∗*^	0.151^*∗*^	0.113	0.121
Concentration	0.187^*∗∗*^	0.252^*∗∗*^	0.215^*∗∗*^	0.227^*∗∗*^	0.281^*∗∗*^	0.204^*∗∗*^	0.319^*∗∗*^	0.283^*∗∗*^	0.328^*∗∗*^	0.276^*∗∗*^	0.273^*∗∗*^	0.136^*∗*^	0.138^*∗*^	0.155^*∗*^
Memory	0.190^*∗∗*^	0.205^*∗∗*^	0.266^*∗∗*^	0.268^*∗∗*^	0.335^*∗∗*^	0.270^*∗∗*^	0.379^*∗∗*^	0.309^*∗∗*^	0.366^*∗∗*^	0.278^*∗∗*^	0.358^*∗∗*^	0.139^*∗*^	0.128^*∗*^	0.145^*∗*^
Headache	0.279^*∗∗*^	0.202^*∗∗*^	0.331^*∗∗*^	0.210^*∗∗*^	0.262^*∗∗*^	0.232^*∗∗*^	0.389^*∗∗*^	0.354^*∗∗*^	0.363^*∗∗*^	0.337^*∗∗*^	0.397^*∗∗*^	0.256^*∗∗*^	0.230^*∗∗*^	0.197^*∗∗*^
Back pain	0.250^*∗∗*^	0.110	0.273^*∗∗*^	0.247^*∗∗*^	0.373^*∗∗*^	0.250^*∗∗*^	0.348^*∗∗*^	0.326^*∗∗*^	0.391^*∗∗*^	0.352^*∗∗*^	0.349^*∗∗*^	0.257^*∗∗*^	0.316^*∗∗*^	0.262^*∗∗*^
Widespread pain	0.178^*∗∗*^	0.052	0.201^*∗∗*^	0.128^∗^	0.185^*∗∗*^	0.195^*∗∗*^	0.302^*∗∗*^	0.260^*∗∗*^	0.281^*∗∗*^	0.208^*∗∗*^	0.244^*∗∗*^	0.152^∗^	0.199^*∗∗*^	0.184^*∗∗*^

^*∗*^ *p* < 0.05; ^*∗∗*^*p* < 0.01.

**Table 2:** Pearson correlation between FMS symptoms and PTSD-related symptoms in the study group (*n* = 242).

	Involuntary sad thoughts	Nightmares	Reexperiencing traumatic event	Anger, sadness, or blame when reminded of event	Physiological feelings in response to remembering traumatic event	Avoiding thoughts about traumatic event	Avoiding activities related to traumatic event	Less interest in important activities	Feeling cut-off or isolated	Feeling emotional dullness	Feeling ones hopes and plans will not be fulfilled	Restlessness	Hypervigilance
Sleep disturbances	0.322^*∗∗*^	0.251^*∗∗*^	0.296^*∗∗*^	0.356^*∗∗*^	0.244^*∗∗*^	0.176^*∗*^	0.260^*∗∗*^	0.317^*∗∗*^	0.315^*∗∗*^	0.277^*∗∗*^	0.322^*∗∗*^	0.476^*∗∗*^	0.298^*∗∗*^
Fatigue	0.265^*∗∗*^	0.235^*∗∗*^	0.207^*∗∗*^	0.324^*∗∗*^	0.200^*∗∗*^	0.165^*∗*^	0.240^*∗∗*^	0.286^*∗∗*^	0.293^*∗∗*^	0.293^*∗∗*^	0.280^*∗∗*^	0.450^*∗∗*^	0.243^*∗∗*^
Joint stiffness	0.171^*∗*^	0.142^*∗*^	0.241^*∗∗*^	0.212^*∗∗*^	0.125	0.189^*∗∗*^	0.186^*∗∗*^	0.176^*∗*^	0.144^*∗*^	0.156^*∗*^	0.296^*∗∗*^	0.377^*∗∗*^	0.249^*∗∗*^
Paresthesia	0.246^*∗∗*^	0.159^*∗*^	0.274^*∗∗*^	0.264^*∗∗*^	0.179^*∗∗*^	0.219^*∗∗*^	0.156^*∗*^	0.222^*∗∗*^	0.161^*∗*^	0.133	0.285^*∗∗*^	0.361^*∗∗*^	0.206^*∗*^
Swelling	0.221^*∗∗*^	0.208^*∗∗*^	0.341^*∗∗*^	0.280^*∗∗*^	0.180^*∗∗*^	0.203^*∗∗*^	0.182^*∗∗*^	0.214^*∗∗*^	0.241^*∗∗*^	0.254^*∗∗*^	0.351^*∗∗*^	0.436^*∗∗*^	0.326^*∗∗*^
IBS	0.171^*∗*^	0.171^*∗*^	0.098	0.208^*∗∗*^	0.157^*∗*^	0.132	0.144^*∗*^	0.175^*∗*^	0.200^*∗∗*^	0.183^*∗∗*^	0.171^*∗*^	0.306^*∗∗*^	0.174^*∗*^
Anxiety	0.392^*∗∗*^	0.292^*∗∗*^	0.357^*∗∗*^	0.401^*∗∗*^	0.300^*∗∗*^	0.277^*∗∗*^	0.331^*∗∗*^	0.320^*∗∗*^	0.355^*∗∗*^	0.289^*∗∗*^	0.334^*∗∗*^	0.510^*∗∗*^	0.321^*∗∗*^
Depression	0.313^*∗∗*^	0.273^*∗∗*^	0.306^*∗∗*^	0.393^*∗∗*^	0.256^*∗∗*^	0.269^*∗∗*^	0.307^*∗∗*^	0.254^*∗∗*^	0.359^*∗∗*^	0.329^*∗∗*^	0.304^*∗∗*^	0.486^*∗∗*^	0.171^*∗*^
Concentration	0.252^*∗∗*^	0.136^*∗*^	0.261^*∗∗*^	0.300^*∗∗*^	0.248^*∗∗*^	0.207^*∗∗*^	0.225^*∗∗*^	0.238^*∗∗*^	0.360^*∗∗*^	0.255^*∗∗*^	0.322^*∗∗*^	0.463^*∗∗*^	0.262^*∗∗*^
Memory	0.241^*∗∗*^	0.122	0.270^*∗∗*^	0.320^*∗∗*^	0.217^*∗∗*^	0.122	0.209^*∗∗*^	0.236^*∗∗*^	0.339^*∗∗*^	0.300^*∗∗*^	0.286^*∗∗*^	0.454^*∗∗*^	0.222^*∗∗*^
Headache	0.212^*∗∗*^	0.115	0.275^*∗∗*^	0.227^*∗∗*^	0.145^*∗*^	0.101	0.192^*∗∗*^	0.219^*∗∗*^	0.300^*∗∗*^	0.260^*∗∗*^	0.279^*∗∗*^	0.329^*∗∗*^	0.304^*∗∗*^
Widespread pain	0.261^*∗∗*^	0.112	0.242^*∗∗*^	0.208^*∗∗*^	0.209^*∗∗*^	0.125	0.148^*∗*^	0.238^*∗∗*^	0.153^*∗*^	0.209^*∗∗*^	0.174^*∗*^	0.265^*∗∗*^	0.341^*∗∗*^
WPI	0.224^*∗∗*^	0.187^*∗∗*^	0.284^*∗∗*^	0.284^*∗∗*^	0.277^*∗∗*^	0.223^*∗∗*^	0.200^*∗∗*^	0.183^*∗∗*^	0.176^*∗*^	0.185^*∗∗*^	0.217^*∗∗*^	0.432^*∗∗*^	0.305^*∗∗*^
SSS	0.250^*∗∗*^	0.151^*∗*^	0.252^*∗∗*^	0.194^*∗∗*^	0.167^*∗*^	0.184^*∗∗*^	0.138^*∗*^	0.173^*∗*^	0.141^*∗*^	0.136^*∗*^	0.235^*∗∗*^	0.347^*∗∗*^	0.291^*∗∗*^
FS	−0.150^*∗*^	−0.096	−0.089	−0.079	−0.128	−0.087	−142^*∗*^	−0.004	−0.058	−0.108	−0.124	−0.212^*∗*^	−0.136

**Table 3:** Comparison between FMS and non-FMS kindergarten teachers regarding work-related performance (*T* test).

	*T* test comparing FMS vs. non-FMS				
	FMS status	*N*	Mean	Std. deviation	Sig. (2-tailed)
Motivation to come to work	FMS	61	6.13	2.649	0.020^*∗*^
	Non-FMS	171	7.08	2.749	

Punctuality of arrival	FMS	61	8.52	2.062	0.110
	Non-FMS	172	8.98	1.859	

Absence from work	FMS	61	7.79	2.715	0.006^*∗∗*^
	Non-FMS	173	8.72	2.093	

Leaving work early	FMS	61	8.59	2.698	0.005^*∗∗*^
	Non-FMS	171	9.44	1.684	

Work performance	FMS	61	8.28	1.572	0.071
	Non-FMS	172	8.69	1.511	

Efforts to raise interest and curiosity among pupils	FMS	61	8.48	1.679	0.060
	Non-FMS	172	8.90	1.446	

Conducting class to one's own satisfaction	FMS	61	7.13	2.148	0.002^*∗∗*^
	Non-FMS	172	7.98	1.679	

To what extent is your work influenced by health issues	FMS	61	8.84	1.368	0.372
	Non-FMS	172	9.03	1.533	

Relationship with pupils	FMS	61	9.31	1.057	0.177
	Non-FMS	173	9.52	1.026	

Caring for pupils	FMS	61	9.25	1.027	0.007^*∗∗*^
	Non-FMS	173	9.62	0.872	

**Table 4:** Comparison of posttraumatic symptoms between kindergarten teachers with and without FMS.

	FMS status	*N*	Mean	Std. deviation	Sig. (2-tailed)
Involuntary sad thoughts	FMS	59	0.92	0.896	0.000^*∗∗*^
	Non-FMS	161	0.45	0.707	

Nightmares related to traumatic event	FMS	58	0.64	0.788	0.002
	Non-FMS	160	0.31	0.616	

Reexperiencing traumatic event	FMS	57	0.58	0.801	0.000^*∗∗*^
	Non-FMS	151	0.23	0.556	

Anger, sadness, or blame when reminded of event	FMS	57	0.93	0.842	0.012^*∗*^
	Non-FMS	159	0.60	0.850	

Physiological feelings in response to remembering traumatic event	FMS	58	0.81	0.888	0.019^*∗*^
	Non-FMS	153	0.50	0.820	

Avoiding thoughts about traumatic event	FMS	58	0.69	0.922	0.071
	Non-FMS	150	0.47	0.739	

Avoiding activities related to traumatic event	FMS	58	0.59	0.838	0.157
	Non-FMS	154	0.41	0.797	

Less interest in important activities	FMS	58	0.95	1.050	0.006^*∗∗*^
	Non-FMS	151	0.55	0.869	

Feeling cut off or isolated	FMS	58	0.88	0.919	0.054
	Non-FMS	154	0.61	0.895	

Feeling emotional dullness	FMS	57	0.81	0.934	0.011^*∗*^
	Non-FMS	153	0.46	0.835	

Hypervigilance	FMS	40	0.88	0.966	0.000^*∗∗*^
	Non-FMS	98	0.21	0.542	

## Data Availability

The data used to support the findings of this study are included within the article.
